# Association of Treatment With 5α-Reductase Inhibitors and Prostate Cancer Mortality Among Older Adults

**DOI:** 10.1001/jamanetworkopen.2019.13612

**Published:** 2019-10-18

**Authors:** Abhishek Kumar, Vinit Nalawade, Paul Riviere, Reith R. Sarkar, J. Kellog Parsons, James D. Murphy, Brent S. Rose

**Affiliations:** 1 Department of Radiation Medicine and Applied Sciences, University of California, San Diego, La Jolla; 2 Department of Urology, University of California, San Diego, La Jolla

## Abstract

This cohort study examines the association between use of 5α-reductase inhibitors and prostate cancer mortality among US Medicare beneficiaries.

## Introduction

5α-reductase inhibitors (5-ARIs) are used to treat benign prostatic enlargement, a common condition causing urinary outflow obstruction.^1^ They also reduce prostate-specific antigen (PSA) by approximately 50%.^2^ Our group has recently published that among US military veterans, 5-ARIs are associated with delays in prostate cancer (PC) diagnoses, higher grade and stage at presentation, and worse PC-specific mortality (PCSM), presumably because of misinterpreted PSA values.^3^ We hypothesized that these results are generalizable to the broader US population.

## Methods

This cohort study followed the Strengthening the Reporting of Observational Studies in Epidemiology (STROBE) reporting guideline. The study was considered exempt from review by the University of California, San Diego institutional review board because it used deidentified data. Data analysis was performed from February 1, 2019, through April 30, 2019.

We used the Surveillance, Epidemiology, and End Results Program–Medicare linked database and identified patients with stage I to IV PC and known PSA level at diagnosis between January 1, 2008, and December 31, 2013; no missing covariate information; and Medicare Part D coverage. Patients were required to have at least 1 year of prediagnosis Medicare enrollment for comorbidity assessment. All patients were followed up until death or December 31, 2015.

We extracted demographic and comorbidity variables, prescription drug information, and tumor-level variables. We defined 5-ARI use as any prescription of finasteride or dutasteride at least 6 months before a PC diagnosis. We doubled the PSA level for 5-ARI users per the Reduction by Dutasteride of Prostate Cancer Events (REDUCE) trial.^4^ We did not include in our models staging or treatment information as covariates because they are on the causal pathway between exposure and outcome.^4^

We tested for differences in covariates between exposure groups using χ^2^ and Wilcoxon rank sum tests. We used multivariable Fine-Gray competing risk regression models to obtain estimates of PCSM and noncancer mortality accounting for competing risk of death. We used multivariable Cox proportional hazards regression models to obtain hazard ratio (HR) estimates of all-cause mortality (ACM). Statistical tests were 2-sided, with *P* < .05 considered statistically significant, and were conducted with SAS statistical software version 9.4 (SAS Institute Inc) and R statistical software version 3.5.1 (R Project for Statistical Computing).

## Results

The final cohort included 30 313 patients, with median (interquartile range) follow-up of 3.75 (2.33-5.25) years. A total of 2373 patients (7.83%) were prescribed 5-ARIs at least 6 months before PC diagnosis, with a median (interquartile range) treatment duration of 2.46 (1.45-3.74) years. The 4-year cumulative incidence of death from PC was 5.3% in 5-ARI users and 2.8% in nonusers.

Compared with participants who did not receive 5-ARI, those who did were more likely to present with disease that was high grade (Gleason score 8-10) (18% vs 29%, respectively; difference, 12%; 95% CI, 9%-13%; *P* < .001), high risk (28% vs 38%; difference, 9%; 95% CI, 8%-11%; *P* < .001), clinically node positive (2% vs 3%; difference, 1%; 95% CI, 0.6%-2%; *P* < .001), and clinically metastatic (2% vs 3%; difference, 1%; 95% CI, 0.5%-2%; *P* < .001). Furthermore, median (interquartile range) adjusted PSA level at diagnosis was significantly higher in 5-ARI users compared with nonusers (14.2 [9.0-24.0] ng/mL vs 6.6 [4.8-10.2] ng/mL; difference, 7.6 ng/mL; 95% CI, 7.0-8.2 ng/mL; *P* < .001 [to convert to micromoles per liter, multiply by 1.0]) (Table 1).

**Table 1.** Baseline Patient Characteristics at Prostate Cancer Diagnosis

Characteristic	No. (%)		*P* Value
	No 5-ARI Use (n = 27 940)	5-ARI Use (n = 2373)	
Race			
Nonblack	25 171 (90)	2208 (93)	<.001
Black	2769 (10)	165 (7)	
Age, median (IQR), y	72 (69-76)	74 (71-79)	<.001
Age, y			
66-70	8215 (29)	416 (18)	<.001
71-74	10 312 (37)	820 (35)	
75-79	5961 (21)	610 (26)	
≥80	3452 (12)	527 (22)	
Charlson Comorbidity Index score			
0	16 226 (58)	1243 (52)	<.001
≥1	11 714 (42)	1130 (48)	
Census tract–level poverty rate			
≥20%	5048 (18)	456 (19)	.17
<20%	22 892 (82)	1917 (81)	
Married			
No	8616 (31)	797 (34)	.01
Yes	19 324 (69)	1576 (66)	
α-Blocker^a^			
No	24 599 (88)	1032 (43)	<.001
Yes	3341 (12)	1341 (57)	
Statin^a^			
No	18 336 (66)	1011 (43)	<.001
Yes	9604 (34)	1362 (57)	
Nonaspirin NSAID^a^			
No	23 252 (83)	1518 (64)	<.001
Yes	4688 (17)	855 (36)	
Year of diagnosis			
2008-2010	14 942 (53)	900 (38)	<.001
2011-2013	12 998 (47)	1473 (62)	
PSA level at diagnosis, median (IQR), ng/mL	6.6 (4.8-10.2)	7.1 (4.5-12.0)	<.001
Adjusted PSA level at diagnosis, median (IQR), ng/mL^b^	6.6 (4.8-10.2)	14.2 (9.0-24.0)	<.001
Log PSA at diagnosis, mean (SD), log ng/mL	1.99 (0.74)	2.01 (0.87)	.37
Log adjusted PSA at diagnosis, mean (SD), log ng/mL	1.99 (0.74)	2.70 (0.87)	<.001
Gleason score			
NA	1916 (7)	166 (7)	<.001
≤7	20 871 (75)	1525 (64)	
≥8	5153 (18)	682 (29)	
Risk group^c^			
Low	7918 (28)	561 (24)	<.001
Intermediate	12 095 (43)	917 (29)	
High	7927 (28)	895 (38)	
Clinical T stage			
1-2	25 925 (90)	2156 (91)	.63
3-4	2645 (10)	217 (9)	
Clinical N stage			
0	27 450 (98)	2303 (97)	<.001
1	490 (2)	70 (3)	
Clinical M stage			
0	27 349 (98)	2296 (97)	<.001
1	591 (2)	77 (3)	
Hormone therapy			
No	18 870 (67)	1388 (58)	<.001
Yes	9070 (33)	985 (42)	
Radiation therapy			
No	11 504 (41)	991 (42)	.59
Yes	16 436 (59)	1382 (58)	
Surgery			
No	17 844 (64)	1521 (64)	.84
Yes	10 096 (36)	852 (36)	

Abbreviations: IQR, interquartile range; NA, not available; NSAID, nonsteroidal anti-inflammatory drug; PSA, prostate-specific antigen; 5-ARI, 5α-reductase inhibitor.

SI conversion factor: To convert PSA to μg/L, multiply by 1.0.

^a^ Medication use was defined as at least 1 prescription filled within the year before prostate cancer diagnosis.

^b^ To obtain adjusted PSA level, we multiplied the PSA for 5-ARI users by 2 to account for PSA suppression.

^c^ Low risk included patients with PSA level less than 10 ng/mL and Gleason group 6 and clinical T stage 1 or 2A; intermediate risk, PSA level greater than or equal to 10 ng/mL and PSA level less than or equal to 20 ng/mL or Gleason group of 7 or clinical T stage 2B or 2C; and high risk, PSA level greater than 20 ng/mL or Gleason group 8 or greater or clinical T stage greater than or equal to 3A.

Use of 5-ARIs was associated with an increased risk of PCSM (subdistribution HR, 1.38; 95% CI, 1.10-1.73; *P* = .005) and ACM (HR, 1.15; 95% CI, 1.01-1.30; *P* = .04). There were no differences in noncancer mortality (subdistribution HR, 1.06; 95% CI, 0.91-1.23; *P* = .47) (Table 2).

**Table 2.** Prostate Cancer–Specific, Noncancer, and All-Cause Mortality

Characteristic	Prostate Cancer–Specific Mortality		Noncancer Mortality		All-Cause Mortality	
	HR (95% CI)	*P* Value	HR (95% CI)	*P* Value	HR (95% CI)	*P* Value
5-ARI, yes vs no	1.38 (1.10-1.73)	.005	1.06 (0.91-1.23)	.47	1.15 (1.01-1.30)	.04
α-Blocker, yes vs no	1.13 (0.94-1.38)	.17	1.30 (1.16-1.45)	<.001	1.24 (1.14-1.38)	<.001
Statin, yes vs no	1.07 (0.92-1.23)	.39	1.01 (0.92-1.11)	.82	1.03 (0.95-1.11)	.52
Nonaspirin NSAID, yes vs no	1.04 (0.87-1.25)	.67	1.03 (0.92-1.15)	.62	1.03 (0.94-1.14)	.53
Charlson Comorbidity Index score, ≥1 vs 0	1.27 (1.11-1.46)	<.001	2.80 (2.56-3.06)	<.001	2.23 (2.07-2.40)	<.001
Married, yes vs no	0.72 (0.63-0.82)	<.001	0.63 (0.58-0.69)	<.001	0.65 (0.61-0.70)	<.001
Age, y						
66-70	1 [Reference]		1 [Reference]		1 [Reference]	
71-74	1.37 (1.09-1.71)	.01	1.31 (1.15-1.49)	<.001	1.33 (1.18-1.48)	<.001
75-79	2.38 (1.91-2.97)	<.001	1.98 (1.73-2.26)	<.001	2.08 (1.86-2.33)	<.001
≥80	5.57 (4.51-6.89)	<.001	4.54 (3.99-5.15)	<.001	4.80 (4.31-5.36)	<.001
Census tract–level poverty rate, ≥20% vs <20%	1.43 (1.21-1.67)	<.001	1.50 (1.36-1.65)	<.001	1.48 (1.36-1.61)	<.001
Year of diagnosis, 2011-2013 vs 2008-2010	0.97 (0.83-1.13)	.67	0.92 (0.83-1.03)	.14	0.94 (0.86-1.02)	.14
Nonblack race vs black race	0.77 (0.63-0.95)	.02	0.76 (0.67-0.85)	<.001	0.76 (0.68-0.84)	<.001

Abbreviations: HR, hazard ratio; NSAID, nonsteroidal anti-inflammatory drug; 5-ARI, 5α-reductase inhibitor.

## Discussion

This cohort study found that 5-ARI users presented with higher adjusted PSA levels and PC disease burden. They also had worse PCSM and ACM, but not worse noncancer mortality. These results are consistent with our recently published findings^3^ that observed that among US veterans, 5-ARI use was associated with worse PCSM (subdistribution HR, 1.39; 95% CI, 1.27-1.52; *P* < .001) and ACM (HR, 1.10; 95% CI, 1.05-1.15; *P* < .001). Like other studies,^3,4,5,6^ we found that comorbidities, unmarried status, old age, low income, and black race were risk factors for PCSM, which adds validity to our findings. One study limitation included the possibility of misclassification bias, in which 5-ARIs were not used as prescribed.

Our results suggest a need for increased awareness of 5-ARI–induced PSA suppression, clearer guidelines for early PC detection, and systems-based mechanisms to help improve care for men using 5-ARIs.
